# Translating the Dietary Approaches to Stop Hypertension (DASH) Diet for Use in Underresourced, Urban African American Communities, 2010

**DOI:** 10.5888/pcd10.120088

**Published:** 2013-01-10

**Authors:** Melicia C. Whitt-Glover, Jaimie C. Hunter, Capri G. Foy, Sara A. Quandt, Mara Z. Vitolins, Iris Leng, Lyndsey M. Hornbuckle, Kara A. Sanya, Alain G. Bertoni

**Affiliations:** Author Affiliations: Jaimie C. Hunter, Gillings School of Global Public Health, The University of North Carolina at Chapel Hill, Chapel Hill, North Carolina; Capri G. Foy, Sara A. Quandt, Iris Leng, Alain G. Bertoni, Maya Angelou Center for Health Equity and Division of Public Health Sciences, Wake Forest University Health Sciences, Winston-Salem, North Carolina; Mara Z. Vitolins, Wake Forest University Health Sciences, Winston-Salem, North Carolina; Lyndsey M. Hornbuckle, Kennesaw State University, Kennesaw, Georgia; Kara A. Sanya, Pharmaceutical Product Development, Inc, Durham, North Carolina.

## Abstract

**Introduction:**

Randomized trials have demonstrated the effectiveness of the Dietary Approaches to Stop Hypertension (DASH) program for lowering blood pressure; however, program participation has been limited in some populations. The objective of this pilot study was to test the feasibility of using a culturally modified version of DASH among African Americans in an underresourced community.

**Methods:**

This randomized controlled pilot study recruited African Americans in 2 North Carolina neighborhoods who had high blood pressure and used fewer than 3 antihypertension medications. We offered 2 individual and 9 group DASH sessions to intervention participants and 1 individual session and printed DASH educational materials to control participants. We collected data at baseline (March 2010) and 12 weeks (June 2010).

**Results:**

Of 152 potential participants, 25 were randomly assigned to either the intervention (n = 14) or the control (n = 11) group; 22 were women, and 21 were educated beyond high school. At baseline, mean blood pressure was 130/78 mm Hg; 19 participants used antihypertension medications, and mean body mass index was 35.9 kg/m^2^. Intervention participants attended 7 of 9 group sessions on average. After 12 weeks, we observed significant increases in fruit and vegetable consumption and increases in participants’ confidence in their ability to reduce salt and fat consumption and eat healthier snacks in intervention compared with control participants. We found no significant decreases in blood pressure.

**Conclusion:**

Implementation of a culturally modified, community-based DASH intervention was feasible in our small sample of African Americans, which included people being treated for high blood pressure. Future studies should evaluate the long-term effect of this program in a larger sample.

## Introduction

Hypertension is a prevalent risk factor for chronic disease (1). National data suggest that 31% of American adults have hypertension (2), and 31% have prehypertension (3). Rates, including rates of uncontrolled hypertension, are higher among African Americans than among whites (2). Dietary Approaches to Stop Hypertension (DASH) is a carbohydrate-rich eating plan that emphasizes increasing consumption of fruits, vegetables, and low-fat dairy products and reducing consumption of saturated fat, total fat, and cholesterol by decreasing consumption of red meat, sweets, and added sugars (4). Randomized controlled trials (RCTs) show DASH to be effective in lowering blood pressure in African Americans (4–9).

Despite the known benefits of DASH and wide availability of free DASH materials, the average US diet does not conform to DASH recommendations (10). Barriers to DASH adherence may include cultural preferences, limited availability and perceived higher cost of DASH foods in underresourced communities, and perceived lack of time for preparing DASH meals (11,12). Limited research is available on the feasibility, acceptability, and effect of DASH among hypertensive or prehypertensive African Americans in community-based settings. The objective of this pilot study was to test the feasibility of using a culturally modified version of DASH among African Americans in an underresourced community. A secondary objective was to determine effect sizes to design an RCT to be implemented in a larger sample.

## Methods

We used a single-cohort RCT design to test the feasibility of our culturally tailored DASH materials and intervention strategies. We examined whether we would be able to achieve a participation rate that compares well with those of other DASH studies and whether our sessions would be well attended. As a secondary study goal, we also evaluated the expected effect size for changes in blood pressure and other laboratory and anthropometric measures, changes in behavior, and confidence in ability to change eating habits, to be used for planning for a larger trial to test the culturally tailored DASH intervention. All procedures were approved by the Wake Forest School of Medicine Institutional Review Board, and the study was registered in clinicaltrials.gov (no. NCT00964483). The 12-week intervention was conducted from March 2010 through June 2010.

### Study setting

We conducted this study in 2 primarily urban, financially underresourced neighborhoods (zip codes 27101 and 27105) in Forsyth County, North Carolina. Compared with the county, the 2 zip code areas have a lower median household income ($29,332 in 27101 and $38,307 in 27105 vs $46,749 in county), a greater proportion of residents earning less than $50,000 annually (72% in 27101 and 62% in 27105 vs 52.8% in county), and higher unemployment rates (8.4% in 27101 and 27105 vs 7.7% in county) (13–15). Both zip code areas are predominantly African American (51% in 27101 and 61.1% in 27105 vs 27.1% in county). Healthy foods are less available in these zip code areas than in higher-income zip code areas in the same county (14).

### Study participants

We recruited participants through mass media advertisements, mailings to people identified on existing lists and by community contacts, and referrals from community partners. Eligibility criteria were 1) self-identifying as African American; 2) being a minimum age of 21 years; 3) self-reporting a primary residence in target zip codes; 4) having a systolic blood pressure of 120 mm Hg to 159 mm Hg, diastolic blood pressure of 80 mm Hg to 99 mm Hg, or both; 5) taking fewer than 3 antihypertensive medications; 6) willing to accept random assignment; and 7) willing to attend intervention sessions at a centralized location. We excluded people who had diabetes, renal disease, heart failure, alcohol or substance abuse, dementia, or schizophrenia. We also excluded underweight people (body mass index [BMI] ≤18.5 kg/m^2^) because the DASH diet could cause weight loss and morbidly obese people (BMI ≥45.0 kg/m^2^), for whom a weight-loss diet and/or pharmacologic or surgical intervention would be indicated. After a brief telephone interview to determine eligibility, we invited potential participants to an information session. People interested in the study and qualified on the basis of blood pressure readings taken at the information session provided informed consent and were scheduled to visit the Wake Forest University General Clinical Research Center, where they were assessed for height and weight, blood pressure, and medical history through standardized protocols. Participants also had a fasting blood draw to obtain measures of glucose and creatinine. People still eligible and wishing to participate had a second visit, during which they were randomly assigned to either the intervention or control group and completed additional questionnaires. The 12-week recruiting and screening process took place from January 2010 through March 2010.

### Intervention

The modified 12-week DASH intervention used materials from the National Heart, Lung, and Blood Institute (NHLBI) (16) and the PREMIER trial (9), used in previous DASH trials. We consulted the Body and Soul program (17) for examples of culturally appropriate foods and dietary practices for African Americans. We conducted focus groups with 30 African Americans from the 2 neighborhoods to understand perceptions about healthy eating, the local food environment, and existing DASH materials. We also conducted objective assessments of 54 stores and 114 restaurants in the target neighborhoods to determine whether residents had access to healthy food (11). These data were used to modify the DASH intervention.

The modified intervention was informed by social cognitive theory (SCT), which posits that engaging in behavior change requires that the social and physical environments support that change (18). Observational learning (modeling) is a major concept of the theory; DASH participants engaged with peers to learn about following a new eating plan, and intervention leaders modeled the desired eating plans and cooking techniques. Participants were given informational binders tailored to the program to facilitate grocery shopping, budget management, and meal planning. They learned to regulate their eating patterns by setting goals for themselves and securing the social support needed to help them sustain gains made from program participation. A core tenet of SCT is that participants build self-efficacy for following a meal plan like DASH to help regulate their blood pressure.

The modified DASH materials were designed to increase participants’ knowledge of community food sources and DASH benefits and to increase skills for preparing meals in accordance with DASH. The targeted behavioral goals were to increase dietary consumption to 9 to 12 servings of fruits and vegetables per day and 2 to 3 servings of low-fat dairy products per day and to decrease fat consumption to less than 25% of total energy, saturated fat consumption to less than 7% of total energy, and sodium consumption to less than 2,300 mg per day (16,19). The modified intervention included 2 individual and 9 group sessions (Box). Individual sessions included one-on-one counseling with the study physician (A.G.B.) and a registered dietitian (L.M.H.) or lifestyle counselor (J.C.H.). Individual sessions consisted of feedback to the participant from the study physician and the dietitian or lifestyle counselor on baseline participant data, counseling on blood pressure and personal program goals, and an opportunity for the participant to ask questions before and after group sessions.

**Box. Session Topics for Dietary Approaches to Stop Hypertension (DASH) Feasibility Study, North Carolina, 2010**

**Table:** 

Session	Topic	Activities
**0**	Individual session 1	Review baseline results, learn about goal setting, and set initial study goals
**1**	Welcome to DASH!	Building healthy meals; reading food labels; serving sizes; sodium control
**2**	Eating out/pre-prepared meals	Incorporating fruits and vegetables into meals; identifying hidden sources of sodium
**3**	Budgeting	Eating healthy on a limited budget
**4**	Grocery store tour	Secrets of grocery shopping
**5**	Breakfast	Healthy breakfast options on the DASH eating plan
**6**	Lunch	Healthy lunch options on the DASH eating plan
**7**	Dinner	Healthy dinner options on the DASH eating plan
**8**	Snacks	Healthy snack options on the DASH eating plan
**9**	Ending session	Share DASH-friendly recipes with one another and friends/family members
**10**	Individual session 2	Set goals for long-term behavior maintenance

Group sessions included discussion and focused on selecting foods and preparing meals in accordance with DASH. They were delivered by a dietitian at a local church that had classrooms and a large kitchen. Participants also practiced meal preparation skills in 4 sessions, assisted by a professional chef.

Control participants attended 1 individual counseling session with the study physician and dietitian and received 2 NHLBI booklets on lowering blood pressure and following the DASH diet (16,20). Control participants were not contacted between the baseline and follow-up data collection periods. They were offered a modified version of the intervention at the end of the study.

### Data collection

We collected data at baseline (March 2010) and 12 weeks (June 2010). All measures were collected at both visits. Because our main objective was to test the feasibility of the modified DASH intervention, primary outcomes of interest were process measures, including participant willingness to engage in various aspects of the study and changes in the targeted dietary behaviors that could lead to changes in blood pressure (eg, dietary intake, shopping practices, social support, barriers to healthy eating). We assessed participant willingness to engage in aspects of the study by the number of people who participated in study screening and enrollment and attended study activities, including intervention sessions and data collection visits. We used the Block Fat/Sugar/Fruit/Vegetable Screener (21) to assess participant dietary intake and measured consumption of fat, fruits, vegetables, and fiber during the previous 4 weeks. Data from six 24-hour diet recalls were collected for each participant (3 at baseline and 3 at follow-up) by trained bionutritionists using Nutrition Data System for Research software (University of Minnesota, Minneapolis, Minnesota). We collected the initial recall in person to allow the bionutritionist to assist participants in recalling food consumption through visual aids and prompts. The 2 subsequent recalls were conducted via telephone by the same bionutritionist who conducted the initial recall. Studies have indicated that telephone administration is a valid, low-burden, cost-efficient method of collecting dietary data (22,23).We measured shopping practices and social support for purchasing fruits and vegetables at baseline and follow-up by using validated scales (24). The Eating Habits Confidence questionnaire measured the extent to which participants felt confident that they could consistently motivate themselves for at least 6 months to improve eating habits such as reducing dietary salt, dietary fat, calories, consuming a healthier diet, and adhering to the DASH diet after the study ended. The Social Support and Eating Habits questionnaire measured perceived support from family and friends for the participant’s efforts at attempting to improve eating habits. Weekly self-monitoring logs were used to increase participant awareness of and progress toward dietary goals.

Secondary outcomes of interest, which were examined to determine effect sizes for a larger RCT, included measures of blood pressure, BMI, waist circumference, lipids, and creatinine. All secondary outcomes were measured at baseline and 12 weeks; blood pressure was also measured at week 6 for intervention participants only. Phlebotomy and laboratory measures were performed by trained clinical staff.

### Statistical analysis

We performed all analyses using SAS 9.2 (SAS Institute Inc, Cary, North Carolina). We used *t* tests for continuous variables and χ^2^ tests for categorical variables to compare baseline characteristics of participants in the control and intervention groups. Analyses of covariance were used to compare outcomes between intervention and control groups, adjusted for baseline measures. Significance level was set at .05.

## Results

### Primary outcomes

We screened 152 potential participants (Figure). We excluded 78 potential participants via telephone, 33 because of diabetes. We obtained informed consent from 52 potential participants; 25 eligible men and women were randomly assigned to the intervention or control group of the study (14 intervention, 11 control).

**Figure:**
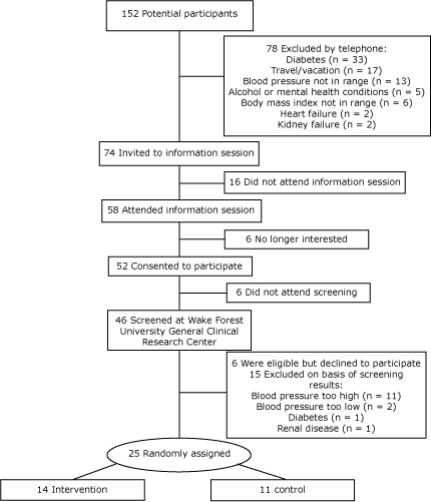
Recruitment, screening, and participation in Dietary Approaches to Stop Hypertension (DASH) Feasibility Study, North Carolina, 2010.

Of the 25 participants, 22 were women, 21 were educated beyond high school, and mean body mass index was 35.9 kg/m^2^. (Table 1). Most were taking blood pressure medications and, on average, blood pressure levels were normal. A significantly greater proportion of control participants than intervention participants were taking blood pressure medications; we observed no other differences between groups at baseline.

**Table 1:** Baseline Characteristics of Participants in the Dietary Approaches to Stop Hypertension (DASH) Feasibility Study, North Carolina, 2010

Characteristic	All (n = 25)	Control (n = 11)	Intervention (n = 14)	*P* Value^a^
**Age, y, mean (SD)**	50.7 (7.9)	49.8 (8)	51.4 (8)	.64
**Men, n**	3	2	1	.40
**Education, n**				
High school degree or less	4	3	1	.44
Some post–high school, no college degree	11	3	8	
College degree or more	10	5	5	
**Body mass index, kg/m^2^, mean (SD)**	35.9 (7.1)	35.9 (7.1)	35.9 (7.3)	.99
**Current smoker, n**	2	2	0	.10
**Blood pressure, mm Hg, mean (SD)**				
Systolic	130.0 (17.3)	127.4 (15.9)	132.0 (18.7)	.52
Diastolic	78.4 (13.2)	76.0 (12.9)	80.4 (13.6)	.42
**Takes blood pressure medication, n**	19	11	8	.01
**Annual household income, n**				
Income <$30,000	6	4	2	.40
Income $30,000–$49,999	7	2	5	
Income >$50,000	12	5	7	
**Number of people supported by household income, mean (SD)**	2.3 (1.2)	2.1 (0.9)	2.5 (1.4)	.42
**Has no medical insurance, n**	3	2	1	.40

Abbreviation: SD, standard deviation.

a Determined by *t* test for continuous variables and χ^2^ test for categorical variables.

Intervention participants attended 7.1 (standard deviation [SD], 2.5) of the 9 group intervention sessions. We found no significant differences overall or within groups between baseline and follow-up for targeted dietary behaviors. Compared with control participants, intervention participants reported significantly greater increases in confidence in their ability to reduce salt and fat consumption and to consume healthier snacks (Table 2). Dietary recall data showed significant increases in mean (SD) servings of fruit (2.3 [1.0]) and vegetables (1.8 [0.6]) among intervention participants compared with control participants.

**Table 2:** Confidence in Ability to Change Eating Habits From Baseline (March 2010) to Follow-up (June 2010), Dietary Approaches to Stop Hypertension (DASH) Feasibility Study, North Carolina, 2010

Factor^a^	All	Control	Intervention	*P* Value^b^	Intervention Effect Estimate, Mean (SD)^c^	*P* Value for Effect^c^
**Confidence in ability to reduce dietary salt**						
Baseline	21.2 (3.8)	21.5 (4)	21.0 (4)	.73	+3.3 (1.4)	.03
Follow-up	21.6 (3.8)	19.8 (5)	23.1 (2)	.03		
**Confidence in ability to reduce dietary fat**						
Baseline	22.1 (2.7)	22.5 (2)	21.9 (3)	.59	+2.2 (0.9)	.02
Follow-up	22.3 (2.6)	21.3 (3)	23.2 (2)	.07		
**Confidence in ability to eat healthier snacks**						
Baseline	19.8 (3)	19.5 (3)	20.0 (3)	.20	+2.8 (1.1)	.02
Follow-up	19.8 (3.5)	18.0 (3)	21.2 (3)	.02		
**Confidence in ability to stick with the DASH diet after study conclusion**						
Baseline	36 (3.7)	36.8 (2)	35.4 (5)	.34	+1.7 (1.1)	.14
Follow-up	36.7 (2.8)	36.1 (3)	37.5 (3)	.20		

a Summary scales were created from a 28-item survey. Original survey items were coded using a Likert scale that ranged from 1 (I know I cannot) to 5 (I know I can). Each factor included 5 to 8 survey items; items were summarized to create a single factor value. Scale ranged from 25 to 40, based on the number of survey items included in the summary scale. Scales for confidence in ability to reduce dietary salt and dietary fat and to increase healthy snack consumption included 5 original survey items. The scale for confidence in the ability to adhere to the DASH diet after study conclusion included 8 original survey items.

b Differences between intervention and control groups determined by analysis of covariance, adjusted for baseline measures.

c Based on linear regression (12 weeks as outcome variable and baseline and randomization arm as predictors).

### Secondary outcomes

We did not observe significant pre–post changes in systolic or diastolic blood pressure (Table 3) in the 2 groups combined or within intervention groups. After 12 weeks, we observed decreases in systolic (−5.6 mm Hg) and diastolic (−5.5 mm Hg) blood pressure in the intervention group compared with control group, but these decreases were not significant (Table 3). Other laboratory and anthropometric measures changed in the expected direction, but differences were not significant overall, within, or between groups. 

**Table 3:** Participant Blood Pressure at Baseline, 6 Weeks, and 12 Weeks, Dietary Approaches to Stop Hypertension (DASH) Feasibility Study, North Carolina, 2010

Blood Pressure	All	Control	Intervention^a^	Effect Size (95% CI)^b^	*P* Value for Effect
**Systolic, mm Hg, mean (SD)**					
Baseline	130.0 (17.3)	127.4 (15.9)	132.0 (18.7)	−5.6 (−19.4 to 8.2)	.41
6 Weeks^c^	129.2 (21.3)	NA	129.2 (21.3)		
12 Weeks	129.8 (18.3)	131.5 (16.8)	128.4 (20.1)		
**Diastolic, mm Hg, mean (SD)**					
Baseline	78.4 (13.2)	76.0 (12.9)	80.4 (13.6)	−5.5 (−13.9 to 2.8)	.18
6 Weeks^c^	79.7 (12.4)	NA	79.7 (12.4)		
12 Weeks	78.3 (10.7)	80.4 (12.2)	76.5 (9.3)		

Abbreviations: CI, confidence interval; SD, standard deviation; NA, not applicable.

a Follow-up blood pressure missing for 1 intervention participant.

b Based on linear regression (12 weeks as outcome variable and baseline and randomization arm as predictors).

c Blood pressure was not measured for control participants at 6 weeks.

## Discussion

Within a limited time, we were able to screen 152 potential participants and randomize 25 (16.4%). Seventy-eight percent of potential participants invited to an information session attended, and 90% of potential participants who attended the session consented to further screening. Of people screened, 54% were eligible for and randomized into our study. Our screening and enrollment rate is similar to the rate of racial/ethnic minority participation in the original DASH study. The original DASH study screened 8,775 people and found 459 eligible (5.2%) (25). Forty-seven percent of people eligible after prescreening in the original DASH study participated in the initial screening visit. Of those who were deemed eligible, 70% participated in an initial 3-week run-in period, during which all participants were fed the control diet. Ninety-one percent of people who completed the run-in period participated in the study, which is higher than our randomization rate; however, the original DASH feeding study had 3 screening visits and an additional 3-week run-in period, increasing the likelihood that participants who remained in the study after such rigorous screening would be eligible. By design, two-thirds of those recruited for the original DASH study were racial/ethnic minorities; 15% of racial/ethnic minority participants who were screened started the initial run-in period before being enrolled (26).

Although not significant, largely because of small sample size, the decreases in blood pressure found in our study are similar to those found in highly controlled feeding studies (studies in which participants are provided all meals). In the original DASH study, 459 participants who had untreated systolic blood pressure of less than 160 mm Hg and untreated diastolic blood pressure of 80 to 95 mm Hg were provided all meals; the study showed a 5.5 mm Hg decrease in mean systolic blood pressure and a 3.0 mm Hg decrease in mean diastolic blood pressure after 8 weeks (after subtracting the effects of the control diet) (4). Another feeding study, focused on sodium modification over 30 days, showed similar decreases in systolic and diastolic blood pressure (5). The PREMIER study, which was not a feeding study, was an RCT to test whether counseling on making several lifestyle changes at once could reduce or control high blood pressure (19). The PREMIER study had 810 participants divided into 3 groups: 1) dietary advice once through a 30-minute education session with a dietitian and no counseling on how to make behavior changes (“advice only”); 2) 18 behavioral counseling sessions during 6 months on how to implement several lifestyle modifications, such as reducing sodium and alcohol consumption, increasing physical activity, and losing weight (“established guidelines” [EG]); or 3) EG and counseling on how to follow the DASH diet (EG + DASH) (9). Hypertension was better controlled in the EG + DASH group than in the advice-only group; blood pressure differences between the EG and EG + DASH groups were not significant. PREMIER was not a feeding study; thus, participants did not adhere to the dietary guidelines as well as participants in previous DASH feeding trials. In our study, participants were taught how to prepare foods in the DASH eating plan; they reported increases in their fruit and vegetable consumption and in their confidence that they could reduce salt and fat consumption and consume healthy snacks. As a result, we achieved decreases in blood pressure similar to those observed in the DASH feeding studies, which highlights the potential for translatability and sustainability of our model. 

Although feeding studies and carefully controlled clinical trials have shown the positive effect of DASH on blood pressure, DASH data from community-based trials are limited. The translation of DASH into community-based settings has shown that blood pressure decreased in participants who attended at least 6 of 8 sessions (27) and adherence to the DASH diet increased when positive messages were associated with dietary goals (28). The percentage of group sessions attended by participants in our study was similar to the percentage attended by participants in previous community-based DASH trials, further demonstrating the feasibility of our model. The Body and Soul dietary intervention conducted in African American churches encouraged consumption of 5 servings of fruits and vegetables daily through church-wide nutrition activities, self-help materials, and motivational interviewing (17). Consistent with SCT concepts, the group-mediated format of the Body and Soul sessions promoted modeling, peer support and encouragement, and strategies to promote self-awareness, self-monitoring, and goal setting for healthy eating. Body and Soul demonstrated increased fruit and vegetable consumption by about 1 serving per day relative to a control intervention; however, the Body and Soul intervention did not evaluate blood pressure or hypertension. We incorporated Body and Soul content in our DASH materials and showed slightly higher — and significant — improvements in self-reported fruit (+2.3 servings) and vegetable (+1.8 servings) consumption in the intervention group compared with the control group.

Another study of DASH among African Americans highlighted the importance of practical problem solving and knowledge acquisition to increase adherence to DASH (29). Our study was largely informed by SCT and emphasized the promotion of self-regulatory skills for healthy nutrition. Also, where possible, we worked with participants to identify solutions to barriers. For example, to address the issue of potentially negative reactions to DASH among family members, participants rehearsed responses to unsupportive family members and identified people who would support their efforts. Family members were invited to the final group session to share in the meal and to discuss the effect of the intervention on the household. Overwhelmingly, family members who attended the session were pleased with and supportive of changes. Our study did, indeed, show positive changes in and increased confidence for preparing and eating foods in accordance with DASH.

Our study had several limitations. It had a small sample size. A larger sample might have shown significant differences between intervention and control groups for blood pressure and other measures. Our study was conducted among African Americans in a primarily urban community in North Carolina, which could limit its generalizability. We had difficulty recruiting eligible participants because the sample was limited to African American adults who had hypertension but not diabetes. Of 152 potential participants, 33 were excluded because of diabetes. Given the high prevalence of multiple chronic conditions among African Americans, future studies should identify methods for translating DASH in this population. Studies evaluating the DASH eating plan among people who have diabetes have noted an inverse association between adherence to DASH and measures of blood glucose and cholesterol levels (30). Additional research is needed to confirm these findings. We did not include objective measures of consumption of foods and nutrients associated with DASH (eg, urinary potassium excretion), and dietary consumption was based on self-report. The study was not powered to detect significant differences in laboratory or anthropometric measures; however, the data provide information about expected effect sizes that can be used to plan a larger randomized trial.

Among adults who reported hypertension in the 1999–2004 National Health and Nutrition Examination Survey, only 22% reported eating a diet compatible with DASH; African Americans were 29% less likely than other population subgroups to report a DASH-compatible diet (31). Further efforts to promote DASH in communities are needed. Our study demonstrated the feasibility of a community-based, culturally modified dietary intervention among African Americans in a primarily urban, financially underresourced area, including people being treated for high blood pressure. This modified DASH intervention should be evaluated over a longer period in a larger sample.
